# Immunogenicity and Safety of a Third SARS-CoV-2 Vaccine Dose in Patients With Multiple Sclerosis and Weak Immune Response After COVID-19 Vaccination

**DOI:** 10.1001/jamaneurol.2021.5109

**Published:** 2022-01-24

**Authors:** Marton König, Hilde Marie Torgauten, The Trung Tran, Trygve Holmøy, John Torgils Vaage, Fridtjof Lund-Johansen, Gro Owren Nygaard

**Affiliations:** 1 Department of Neurology, Oslo University Hospital, Oslo, Norway; 2 Neuro-SysMed, Department of Neurology, Haukeland University Hospital, Bergen, Norway; 3 Department of Immunology, Oslo University Hospital, Oslo, Norway; 4 Department of Neurology, Akershus University Hospital, Oslo, Norway

## Abstract

This cohort study investigates the immunogenicity and safety of a third SARS-CoV-2 vaccine dose in patients with multiple sclerosis who had a weak immune response to COVID-19 vaccination.

Approximately 80% of all patients with multiple sclerosis (MS) treated with anti-CD20 therapy or fingolimod have weak humoral immune responses after 2 doses of messenger RNA (mRNA) COVID-19 vaccines.^1,2,3^ The outcome and safety of a third vaccine dose in this group is largely unknown. We reported results from a study designed to assess the immunogenicity and safety of a third dose of mRNA COVID-19 vaccine in patients with MS who were treated with anti-CD20 therapy or fingolimod.

## Methods

Patients from 3 university hospitals who were enrolled beginning on March 23, 2021, in an ongoing observational cohort study on immune responses after 2 doses of mRNA vaccines^2^ were offered a third dose (either BNT162b2 [Pfizer-BioNTech] or mRNA-1273 [Moderna]) from July 7, 2021, based on the following inclusion criteria: (1) signed, electronically obtained informed consent and (2) SARS-CoV-2 spike and receptor-binding domain (RBD) IgG less than 70 arbitrary units (AU) after full vaccination. Exclusion criteria were adverse reactions to previous mRNA vaccination, pregnancy, and ongoing acute infection. Antibodies to full-length spike protein from SARS-CoV-2 and the RBD were measured using an in-house bead-based flow cytometric assay^4^ in all included patients 3 to 12 weeks after full vaccination and 3 to 5 weeks after revaccination. Postimmunization IgG titers were used as a correlate of protection.^5^ Reduced immunity was assumed in individuals with IgG less than 70 AU corresponding to a lower level than that found in 99% of healthy vaccinated individuals.^2^ IgG levels less than 5 AU were defined as no antibody response, whereas IgG levels between 5 and 70 AU were defined as a weak antibody response. Background variables were acquired through a digital questionnaire completed by all patients and from patient journals. Information regarding adverse effects was collected 3 to 5 weeks after revaccination. Information regarding COVID-19 vaccines was extracted from the Norwegian Immunization Registry. This study was approved by the Regional Ethical Committee and the Norwegian Medicines Agency and followed the Strengthening the Reporting of Observational Studies in Epidemiology (STROBE) guidelines. Continuous and categorical variables were compared using the Mann-Whitney *U* test. A 2-sided *P *value < .05 was considered statistically significant. Correlations were assessed using Spearman ρ. Statistical analyses were performed using SPSS, version 26 (IBM).

## Results

Of 175 invited patients with MS, 130 (74.2%) met the inclusion criteria. The median (IQR) patient age was 47.5 (40.6-56.0) years, and the study included 97 women (74.6%) and 33 men (25.4%). A total of 100 patients (76.9%) received rituximab, 1 patient (0.8%) received ocrelizumab, and 29 patients (22.3%) received fingolimod. All patients underwent revaccination and antibody testing before October 1, 2021 (Table).

**Table.** Clinical, Demographic, and Vaccination-Specific Variables of Patients With Multiple Sclerosis Who Underwent Revaccination

Study population	No. (%)		
	All patients (N = 130)	Anti-CD20 (n = 101)	Fingolimod (n = 29)
Sex			
Women	97 (74.6)	80 (79.2)	17 (58.6)
Men	33 (25.4)	21 (20.8)	12 (41.4)
Age, median (IQR), y	47.5 (40.6-56.0)	47.2 (40.0-55.7)	48.9 (41.8-56.9)
Disease duration, median (IQR), y	6.7 (2.6-13.6)	5.1 (2.6-11.6)	14.1 (7.6-18.6)
Disability by EDSS score, median (IQR)	2.0 (1.0-3.1)	2.0 (1.0-3.1)	2.0 (1.0-3.3)
MS type			
Relapsing-remitting	114 (87.7)	85 (84.2)	29 (100)
Progressive	17 (13.1)	16 (15.8)	0
SARS-CoV-2 RBD IgG titer after full vaccination, AU			
Median (IQR)	3 (2.0-28.8)	3 (2.0-7.0)	5 (2.6-8.5)
Mean (SD)	9.0 (13.6)	8.9 (13.9)	9.2 (12.7)
<5	80 (61.5)	67 (66.3)	13 (44.8)
5-70	50 (38.5)	34 (33.7)	16 (55.2)
>70	0	0	0
SARS-CoV-2 RBD IgG titer after revaccination, AU			
Median (IQR)	6 (3.0-59.3)	4 (2.0-69.5)	13 (4.0-38.0)
Mean (SD)	44.0 (68.8)	49.4 (75.7)	25.1 (29.6)
<5	59 (45.4)	51 (49.5)	8 (27.6)
5-70	44 (33.8)	25 (24.8)	19 (65.5)
>70	27 (20.8)	25 (24.8)	2 (6.9)
Time from last treatment dose to revaccination, median (IQR), mo	NA	4 (2.1-5.6)	NA
Absolute lymphocyte count, median (IQR), cells/mm^3^	1300 (900-1700)	1500 (1200-1800)	550 (425-775)
CD19 B-cell count, median (IQR), cells/mm^3^	NA	0 (0-2.3)	NA
Type of first vaccine dose			
BNT162b2	107 (82.3)	87 (86.1)	20 (69.0)
mRNA-1273	18 (13.8)	10 (9.9)	8 (27.6)
ChAdOx1-S	4 (3.1)	3 (3.0)	1 (3.4)
Type of second vaccine dose			
BNT162b2	111 (85.4)	90 (89.1)	21 (72.4)
mRNA-1273	19 (14.6)	11 (10.9)	8 (27.6)
Time between second vaccine dose and antibody sample, median (IQR), d	39 (23.0-77.0)	53 (25.0-81.5)	28 (21.0-36.8)
Time between second vaccine dose and third vaccine dose, median (IQR), d	84 (71.5-109.0)	91 (73.5-113.5)	77 (67.5-83.0)
Type of third vaccine dose			
BNT162b2	19 (14.6)	12 (11.9)	7 (24.1)
mRNA-1273	111 (85.4)	89 (88.1)	22 (75.9)
Time between third vaccine dose and antibody sample, median (IQR), d	22 (21.0-25.0)	22 (21.0-24.5)	23 (21.0-35.0)
Adverse effects			
Total	75 (57.7)	64 (63.4)	11 (37.9)
Sought medical help	4 (3.1)	4 (4.0)	0
Hospitalization	0	0	0
MS relapse	0	0	0

Abbreviations: AU, arbitrary units; EDSS, Expanded Disability Status Scale; MS, multiple sclerosis; NA, not available; RBD, receptor-binding domain.

After full vaccination, mean (SD) levels of anti–SARS-CoV-2 spike RBD IgG titer were as follows: anti-CD20 group, 8.9 (13.9) AU and fingolimod group, 9.2 (12.7) AU. After revaccination, mean (SD) levels of anti–SARS-CoV-2 spike RBD IgG titer increased significantly in both treatment groups to 49.4 (75.7) AU in the anti-CD20 group (*P* < .001) and to 25.1 (29.6) AU in the fingolimod group (*P* = .006). The proportion of patients with assumed protective humoral immunity (IgG > 70 AU) after revaccination included 25 of 101 patients (24.8%) given anti-CD20 therapy and 2 of 29 patients (6.9%) treated with fingolimod (Figure). Among those with RBD IgG less than 70 AU, compared with those with RBD IgG greater than 70 AU, higher absolute lymphocyte count (mean [SD], 1262 [584] cells/mm^3 ^vs 1508 [560] cells/mm^3^; *P* = .03) and higher CD19 B-cell counts (in patients receiving anti-CD20 therapy: mean [SD], 6 [17] cells/mm^3^ vs 25 [53] cells/mm^3^; *P* = .03) were associated with the development of protective humoral immunity. We found no correlation between antibody responses and time from last anti-CD20 infusion to revaccination (Spearman ρ correlation coefficient, 0.70; *P* = .50) or the cumulative duration of treatment (Spearman ρ correlation coefficient, −0.17; *P* = .09).

**Figure.**
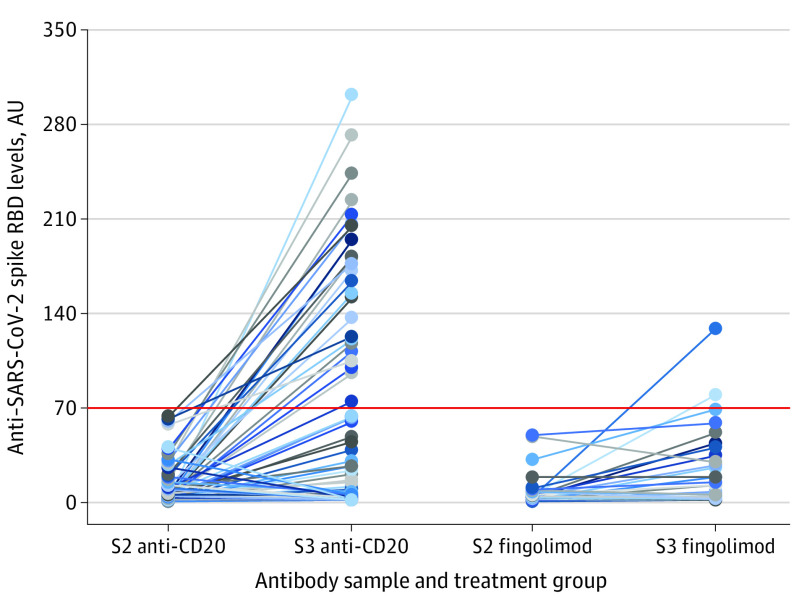
Development of Anti–SARS-CoV-2 Spike Receptor-Binding Domain (RBD) IgG Levels in Patients With Multiple Sclerosis Who Were Treated With Anti-CD20 or Fingolimod and Underwent Revaccination Reduced immunity was assumed in individuals with IgG levels < 70 arbitrary units (AU; red horizontal line) corresponding to a lower level than found in 99% of healthy vaccinated individuals. S2 indicates antibody sample after second vaccine dose; S3, antibody sample after third vaccine dose.

Adverse effects were observed in 64 of 101 patients (63.4%) with MS treated with anti-CD20 therapy and in 11 of 29 patients (37.9%) treated with fingolimod, the most common being transient local pain and fatigue (Table). No patients experienced serious adverse effects after revaccination. The mean (SD) absolute lymphocyte count was higher in patients who reported adverse effects (1410 [594] cells/mm^3^) than in patients who did not report adverse effects (1183 [564] cells/mm^3^; *P* = .03).

## Discussion

The results of this cohort study showed that a third dose of the mRNA COVID-19 vaccine was safe and associated with modestly increased levels of anti–SARS-CoV-2 spike RBD IgG antibodies in patients with reduced protective humoral immunity before reimmunization. A higher absolute lymphocyte count was associated with a better antibody response and more adverse effects, and a higher proportion of patients who were treated with anti-CD20 therapy experienced a better antibody response than patients treated with fingolimod. A 25% increase in the number of patients who experienced seroconversion after revaccination and who were treated with anti-CD20 therapy may be of clinical relevance, as these patients have an approximately 3-fold risk of developing serious COVID-19^6^; therefore, our study results suggest that revaccination of these patients should be considered.

The primary limitation of this study was that it only included assessments of IgG response as a measure of presumed humoral immunity. It is important to note, however, that antibody levels are not fully predictive of protection against infection and that levels lower than the applied cutoff may also be protective. Furthermore, the protective immune response to SARS-CoV-2 also probably depends on T-cell responses.

## References

[1] Achiron A, Mandel M, Dreyer-Alster S, . Humoral immune response to COVID-19 mRNA vaccine in patients with multiple sclerosis treated with high-efficacy disease-modifying therapies. Ther Adv Neurol Disord. 2021;14:17562864211012835. doi:10.1177/17562864211012835 34035836 PMC8072850

[2] König M, Lorentzen AR, Torgauten HM, . Humoral immunity to SARS-CoV-2 mRNA vaccination in multiple sclerosis: the relevance of time since last rituximab infusion and first experience from sporadic revaccinations. J Neurol Neurosurg Psychiatry. 2021;jnnp-2021-327612. doi:10.1136/jnnp-2021-327612 34670844 PMC9763174

[3] Brill L, Rechtman A, Zveik O, . Humoral and T-cell response to SARS-CoV-2 vaccination in patients with multiple sclerosis treated with ocrelizumab. JAMA Neurol. Published online September 23, 2021. doi:10.1001/jamaneurol.2021.3599 34554197 PMC8461553

[4] Holter JC, Pischke SE, de Boer E, . Systemic complement activation is associated with respiratory failure in COVID-19 hospitalized patients. Proc Natl Acad Sci U S A. 2020;117(40):25018-25025. doi:10.1073/pnas.2010540117 32943538 PMC7547220

[5] Earle KA, Ambrosino DM, Fiore-Gartland A, . Evidence for antibody as a protective correlate for COVID-19 vaccines. Vaccine. 2021;39(32):4423-4428. doi:10.1016/j.vaccine.2021.05.063 34210573 PMC8142841

[6] Sormani MP, De Rossi N, Schiavetti I, ; Musc-19 Study Group. Disease-modifying therapies and coronavirus disease 2019 severity in multiple sclerosis. Ann Neurol. 2021;89(4):780-789. doi:10.1002/ana.26028 33480077 PMC8013440

